# Total Urinary Arsenic and Inorganic Arsenic Concentrations and Birth Outcomes in Pregnant Women of Tacna, Peru: A Cross-Sectional Study

**DOI:** 10.1007/s12403-020-00377-2

**Published:** 2020-09-01

**Authors:** Diego Fano-Sizgorich, Cinthya Vásquez-Velásquez, Sandra Yucra, Vanessa Vásquez, Patricio Tokeshi, Julio Aguilar, Claudio Ramírez-Atencio, Dana Boyd Barr, Gustavo F. Gonzales

**Affiliations:** 1 Laboratory of Endocrinology and Reproduction, Universidad Peruana Cayetano Heredia, Av. Honorio Delgado 430, San Martín de Porres, Lima 31, Lima, Peru; 2 Universidad Nacional Jorge Basadre Grohmann, Tacna, Peru; 3 Dirección Regional de Salud, Gobierno Regional de Tacna, Tacna, Peru; 4 Gangarosa Department of Environmental Health, Rollins School of Public Health, Emory University, Atlanta, GA 30322 USA

**Keywords:** Urinary arsenic, Birth outcomes, Arsenic exposure, Tacna

## Abstract

Inorganic arsenic exposure has been linked to the development of several health conditions, including adverse birth outcomes, and around 150 million of people worldwide are exposed to levels above the WHO suggested limit of 10 μg/L. A recent risk assessment in pregnant women of Tacna, of this same population performed by our group, found that 70.25% were exposed to arsenic concentrations in drinking water ≥ 25 μg/L. The present study aimed to evaluate the relationship between prenatal total urinary arsenic (U-tAs) and inorganic arsenic (U-iAs) with adverse birth outcomes. A total of 147 pregnant women from the province of Tacna, Peru, during February–March, 2019, were evaluated for U-tAs and U-iAs exposure during their second trimester of pregnancy, while the birth records of their children were collected from the local hospital. The geometric mean U-tAs was 43.97 ± 25.88 μg/L (P_50_ 22.30, range 5.99–181.94 μg/L) and U-iAs was 5.27 ± 2.91 μg/L. Controlling for maternal age, pre-pregnancy BMI, parity, mother’s education, and newborn sex, no relationship was observed between tertile of U-tAs and the birth outcomes considered, although we found an apparent but statistically non-significant dose–response relationship for small for gestational age 2.38% (95% CI 0.003, 0.16), versus 7.32% (95% CI 0.02, 0.21%), and versus 8.57% (95% CI 0.03, 0.25%). This finding requires further evaluation considering other factors such as metabolic arsenic species, additional maternal covariates, and ethnicity.

## Introduction

The World Health Organization (WHO) estimates that about 150 million people in 70 different countries have been consuming water with arsenic (As) levels above the recommendation of 10 µg/L (Brammer and Ravenscroft 2009; WHO 2011). Although Bangladesh and India are considered to have the most concerning exposure levels (Milton et al. 2017), high levels of As have also been found in Latin American countries where it is estimated that 4.5 million people are exposed to levels of inorganic As (iAs) greater than 50 μg/L with the highest levels found in Argentina, Bolivia, Chile, Mexico and Peru (McClintock et al. 2012).

Fetal vulnerability to environmental pollutants, such as air pollutants, and metals including arsenic, is closely related to maternal exposure, which could lead to several health effects at birth and through life (van de Bor 2019). Inorganic As exposure has been linked to the development and progression of several chronic diseases and health conditions, such as skin diseases (Ratnaike 2003), metabolic disorder such as diabetes when exposed to urinary total arsenic (U-tAs) levels of 5.5 μg/L (Park et al. 2016; Singh et al. 2011), and cancer (IARC 2012). Arsenic has the capability to cross the placenta (Concha et al. 1998; Hall et al. 2007) and affect fetal growth and development.

Arsenic exposure has been associated with adverse birth outcomes such as low birth weight in exposures ≥ 50 μg/L in groundwater (Quansah et al. 2015) and preterm birth (Milton et al. 2017), but also some studies do not find association even with high exposure levels in water with median 73 μg/L (Kwok et al. 2006).

In Peru, high levels of arsenic have been found in surface waters, mainly in the regions of Moquegua, Tacna and Lima (Castro De Esparza 2006; George et al. 2014). Because of its location at the head of the Atacama desert, Tacna possesses an abundance of As-rich volcanic rock soil to which its population has been exposed for millennia (Fano et al. 2019a).

We have recently performed a drinking water-based risk assessment (February–March, 2019) on 161 pregnant women in the province of Tacna where we found that water iAs concentrations were ≥ 50 μg/L in 34.8%, 25–50 μg/L in 35.4% and ≤ 10 μg/L in 29.8% of the samples tested, with increasing As levels from the southern city area to the north (Fano et al. 2019b). In the present study, we evaluate the relationship between urinary total arsenic (U-tAs) and inorganic arsenic (U-iAs) with birth weight, birth length, gestational age, preterm birth, small for gestational age (SGA) and large for gestational age (LGA) in this same cohort.

## Material and Methods

### Design

We conducted a cross-sectional observational study, during February–March in 2019, in which urine samples were obtained during the second trimester of pregnancy, and birth outcomes data were obtained after deliveries. The study protocol was reviewed and approved by the Universidad Peruana Cayetano Heredia Institutional Review Board.

### Study Population

The population evaluated was derived from a previous risk assessment study performed by our group in 2019. The study population recruitment process and description can be found in detail elsewhere (Fano et al. 2019b). In brief, the study sample included 161 pregnant women ≤ 24 weeks of gestation who sought regular antenatal care in any of the 16 different health establishments of the province of Tacna.

Other inclusion criteria included women of 18–40 years old that have been living at least for 5 years in the province of Tacna. Of these 161 pregnant women, 147 (91.3%) provided a urine sample for biological evaluation of U-tAs and U-iAs exposure. Mean gestational age at the time of recruitment and urine sampling was 15.7 ± 4.7 weeks.

The province of Tacna has four public health centers capable of attending neonatal delivery: the San Francisco Health Center, the Ciudad Nueva Health Center, the La Esperanza Health Center, and the Hipólito Unanue Hospital. The latter health center is a teaching hospital (Level II-2) which provides specialized and comprehensive health care to the Tacna Region and those referred by other institutions including births requiring a cesarean section or that is in high risk.

### Assessment of Arsenic Exposure

Based on our previous exposure assessment (Fano et al. 2019b), the most present arsenical compound is inorganic arsenic. Pregnant women were asked to not consume fish-based meals at least 3 days prior sampling. Urine samples were self-collected by the pregnant women in a sterile plastic flask after receiving instructions for collection. All pregnant women were ≤ 24 weeks gestation at the moment of sampling.

The urine collected was a “clean catch” after eliminating the first few mL urine from a first morning void. Participants were asked to freeze the sample in their home freezer; the sample was retrieved by our team on the same day of sample collection according to participant availability. After retrieval, the sample was immediately put in a cooler with cooling packs and transported to the Universidad Nacional Jorge Basadre Grohmann biology laboratory where two 2-mL aliquots were separated into cryotubes and temporary stored at − 20 °C.

The samples were sent to the Rollins School of Public Health at Emory University where they were analyzed for total arsenic and individual arsenic species. Urinary total As, iAs^III^ and iAs^V^, were measured in 250 µL aliquots of urine. Unknown samples were prepared concurrently with three blank samples, calibration samples, NIST reference material SRM 1643f, and two levels of quality control samples per analytic run. For urinary total As measurements, samples were digested with nitric acid before dilution with a mixture of internal standards (indium, iridium, lutetium, and rhodium). For speciated analyses, urine samples were diluted with the internal standards then separated using strong anion exchange chromatography using a gradient elution with ammonium carbonate ((NH4)2CO3), ethylenediaminetetraacetic acid disodium salt (Na2EDTA), and methanol. The samples were then analyzed via inductively coupled plasma mass spectrometry (ICP-MS), removing spectral interferences with a collision reaction cell. Concentrations of the target elements were determined from the ratio of the instrument response to the native analyte to the response to the internal standards in the sample, by comparison to the standard curve. NIST SRM 3667 was used as a quality control material.

U-tAs, U-iAs^III^, and U-iAs^V^ levels were adjusted by specific gravity. Specific gravity was determined using an automated refractometer which was calibrated with water prior to use. The limit of detection (LOD) was 0.1 μg/L for U-tAs, U-iAs^III^, and U-iAs^V^; the frequency of detection was 100%. Relative standard deviation in repeated analyses was < 10%. U-iAs was expressed as the sum of U-iAs^III^ and U-iAs^V^.

### Birth Outcome Data

Data on maternal and perinatal outcomes were obtained from the medical records of the Hipólito Unanue Hospital in Tacna which are included in the Perinatal Information System, SIP in Spanish. The data collected included gestational age, birth length, and birth weight which were used to generate the variables preterm birth (PTB) and small for gestational age (SGA) and large for gestational age (LGA). SGA and LGA were defined as birth weight below the 10th percentile or above the 90th percentile, respectively, by gestational age. PTB was defined as a birth occurring < 37 weeks of gestation.

In cases where the pregnant woman did not give birth at this Hospital, the clinical record of the corresponding health center was consulted. In cases where care was provided through a private system or sufficient data were lacking, the participant was asked to provide her child's information, this was the case for 4 participants (2.48% of recruited sample). Only 6 women gave birth in a health establishment different from the hospital, of which 1 was in a private clinic.

### Covariates

The covariates controlled for were maternal age, pre-gestational BMI, parity, and maternal education level, because of their strong association with previous studies on perinatal outcomes (Estrada-Restrepo et al. 2016; Li et al. 2018; Merklinger-Gruchala et al. 2015).

These variables were obtained by survey, based on objective questions and also from the review of maternal electronic clinical record. Other covariates such as type of zone of residence (rural or urban) were not considered since all pregnant women belonged to the urban zone of the city of Tacna.

### Statistical Analysis

The data were analyzed using STATA software version 15.0 for personal computers (College Station, TX 77845). SGA and LGA were variables generated based on a standardized fetal growth chart (Kiserud et al. 2017). The analysis was based only in the 147 pregnant women that provided their urine sample.

U-tAs and U-iAs concentrations (μg/L) were log transformed and then divided into tertile of exposure with continuous (birth weight, gestations age) and categorical (PTB, SGA and LGA) outcome variables tested using a one-way analysis of variance (ANOVA) and Pearson’s Chi-squared test, respectively. Linear (birth weight, gestational age) and logistic (PTB, SGA, LGA) regression models were developed to evaluate the relationship between log-tAs and log-iAs and outcome variables controlling for maternal age, prenatal BMI, parity, mother’s education, and newborn sex; smoking was not considered as cofounder since only 4 women expressed to smoke at least one-week prior the interview. A *p*-value < 0.05 was considered significant.

## Results

The geometric mean of U-tAs concentration in the sampled population was 43.97 μg/L (95% CI 39.8–48.6), range (6.0–181.9 μg/L). Lowest to highest tertiles were ≤ 34.26 μg/L, > 34.26 to ≤ 56.82 μg/L, and > 56.82 μg/L. On the other hand, the U-iAs for the total population of pregnant women was 5.27 μg/L (95% CI 4.42–6.28). We only had 1 case of low birth weight at term, so this birth outcome category was not further considered; this participant’s U-tAs was 80.78 μg/L, belonging to the upper tertile.

Table 1 shows the population characteristics and birth outcomes distribution. We found no significant differences between the exposure tertiles (T1, T2 and T3) for total and inorganic arsenic in urine with respect to perinatal outcomes, although we did observe a non-significant dose–response relationship between SGA and tertile of exposure. Similarly, we did not observe any statistically significant associations between exposure and outcomes in our models (Table 2).

**Table 1:** Population and tertile exposure groups characteristics and birth outcomes summary statistics

	Population	T1	T2	T3	*p*-value
*N*	147	48	49	50	–
U-tAs	52.65 ± 33.31	23.71 ± 6.84	43.94 ± 6.90	88.96 ± 31.20	–
U-iAs	5.27 ± 2.91	2.58 ± 3.73	5.17 ± 1.66	10.49 ± 7.64	–
Log-tAs	3.78 ± 0.61	3.11 ± 0.34	3.77 ± 0.16	4.44 ± 0.31	–
Log-iAs	1.66 ± 1.07	0.43 ± 0.82	1.85 ± 0.27	2.67 ± 0.37	–
Age	28.16 ± 6.08	28.46 ± 5.99	28.06 ± 5.86	27.98 ± 6.46	0.91
BMI	26.55 ± 4.83	27.27 ± 5.85	26.51 ± 3.79	25.89 ± 4.67	0.76
Birth weight	3633.22 ± 472.87	3646.02 ± 404.53	3606.59 ± 521.78	3647.87 ± 489.51	0.89
Birth length	50.60 ± 2.48	50.31 ± 3.21	51.07 ± 1.79	50.55 ± 2.30	0.37
Gestational age	38.80 ± 1.83	38.56 ± 2.55	38.88 ± 1.29	39.03 ± 1.16	0.48
Preterm birth	4.88	2.22	7.14	5.56	0.55
SGA	5.93	2.38	7.32	8.57	0.46
LGA	34.15	35.56	33.33	33.33	0.96

Values are presented as geometric means ± geometric standard deviation. One-way analysis of variance was used to compare birth weight, birth length, and gestational age between tertiles. Pearson’s Chi-squared test was employed to compare preterm birth, SGA and LGA proportion between tertiles

*U-tAs* Urinary total arsenic, *U-iAs* Urinary inorganic arsenic

**Table 2:** Regression analyses between urinary arsenic (Log-tAs and Log-iAs) and birth outcomes

log-tAs	OR (95% CI)		
	Crude	Adjusted	*p*-value
Birth weight†	34.89 (− 94.71, 164.50)	41.57 (− 88.37, 171.51)	0.52
Birth length†	0.11 (− 0.65, 0.86)	0.09 (− 0.66, 0.85)	0.80
Preterm birth‡	1.44 (0.38, 5.50)	1.59 (0.38, 6.61)	0.52
SGA‡	1.003 (0.29, 3.42)	0.93 (0.28, 3.08)	0.90
LGA‡	0.96 (0.52, 1.76)	1.003 (0.54, 1.88)	0.99
log-iAs			
Birth weight†	42.12 (− 31.25, 115.50)	56.39 (− 16.28, 129.07)	0.127
Birth length†	0.07 (− 0.21; 0.36)	0.06 (− 0.22; 0.35)	0.65
Preterm birth‡	1.41 (0.58; 3.42)	1.37 (0.55; 3.41)	0.48
SGA‡	0.76 (0.40; 1.45)	0.62 (0.27; 1.40)	0.25
LGA‡	0.97 (0.70; 1.36)	1.02 (0.71; 1.48)	0.88

Regression coefficient β† (95% CI) and OR‡ (95% CI) crude and adjusted for mother's age, pre-gestational BMI, newborn’s sex, parity, and education. *P*-value presented corresponds to the adjusted model

*SGA* small for gestational age, *LGA* large for gestational age

## Discussion

Tacna is the region with highest As contamination in water in Peru (Fano et al. 2019a). The geometric mean U-tAs in our population was 44.97 μg/L and U-iAs was 5.37 ug/L. In the Person’s Chi-squared test, we found no association between urinary tAs tertile of exposure and the different birth outcomes, although a non-significant increasing trend for SGA was observed (T1 2.38%, T2 7.32%, and T3 8.57%, *p* = 0.46).

The U-tAs levels found in our population are almost five times higher than levels in the adult female US population (CDC 2019), and three times higher than a pregnant women cohort of Arica, Chile (Muñoz et al. 2018). However, compared to an indigenous women cohort from Southeast Bolivia, our U-tAs levels were about 32% lower (De Loma et al. 2019). Similarly, and as expected, our U-tAs levels are much lower than those observed in high-As-contaminated regions such as Bangladesh and India (Milton et al. 2017).

Despite the relatively high levels of U-tAs in 2nd trimester urine in our population, we found no significant association between tertile of U-tAs or log-tAs and birth outcomes. We did observe a trend in SGA with exposure tertile but it was statistically non-significant (*p* = 0.46).

Current evidence linking birth outcomes to prenatal As exposure is inconsistent, two systematic reviews agree that there is concise evidence that increase arsenic exposure is associated with low birth weight, stillbirth and spontaneous abortion but this was mostly found with drinking water as exposure source (Milton et al. 2017; Quansah et al. 2015; Xu et al. 2011), while some recent cohort studies have found increased risk for preterm birth when exposed to drinking water As (Rahman et al. 2018), and SGA with U-tAs (Liu et al. 2018).

Low-level As in water has been linked to birth outcomes in an ecological analysis in Ohio, USA, in which mean arsenic concentrations ranged from 0.5 to 12.2 μg/L, where positive associations with very low birth weight and PTB were observed (Almberg et al. 2017), and according to our previous risk assessment in this same sample, 111 women were exposed to level ≥ 25 μg/L (Fano et al. 2019b). In Romania, drinking water As > 10 μg/L, mean 4.11 μg/L, was linked to birth weight and birth length Z-scores particularly in smokers, with a mean iAs of 4.11 μg/L, based in a sample of 122 pregnant women (Bloom et al. 2016).

In another study, U-iAs, P_50_ 0.3 μg/L, was not associated with birth outcomes such as gestational age, birth length, and birth weight (Gilbert-Diamond et al. 2016). Despite having similar or higher levels of urinary tAs and iAs, a clear relationship with the adverse birth outcomes we studied was not observed. Other birth outcomes that have been linked to As exposure could not be evaluated because of lack of data, such as the negative association found between second trimester U-tAs, mean 40 μg/L, and head circumference in a Taiwanese cohort study of 133 pregnant women (Liao et al. 2018).

An interesting finding in our study was the high LGA prevalence in our population (34.2%) which is almost 6.5 times the national average in Peru (Alves da Cunha et al. 2017). Similarly, our LGA proportion is higher than in most studies that have observed associations between U-tAs and birth outcome (Mullin et al. 2019).

This high prevalence of LGA may obscure any associations with specific birth outcomes in our population. Tacna may display a high prevalence of LGA given its ethnic component; according to the last census in 2017, 33% of Tacna’s population identified themselves as Aymara (INEI 2018), and based in our risk assessment, around 50% of the sample considered themselves as Aymara (Fano et al. 2019b); and as found in other studies, Aymaras are characterized by high birth weights (Rothhammer et al. 2015). This factor should be genetically explored and considered in future studies.

Our study has several strengths and limitations. We were able to measure internal exposure to U-tAs and U-iAs during pregnancy on a population on which water As levels had been previously determined, and evaluated for the first time the association between U-tAs and U-iAs with birth outcomes in this population. However, As does not appreciably persist in the body, being almost fully eliminated through the urine in 3–5 days (Ratnaike 2003), and a single sample during the 2nd trimester may not have accurately captured average exposure during pregnancy. In a recent study, high As exposures during early and late pregnancy were not associated with SGA; however, As at delivery was associated (Mullin et al. 2019).

In another study, 3rd-trimester As exposure was associated with adverse birth outcomes, median 21.08 μg/L, including birth weight, birth length and SGA (Liu et al. 2018), thus the timing of our sample collection may have obscured any true associations. Another limitation of our study is the lack of arsenic speciated results. In some studies, outcomes can be better observed when evaluating specific As species in urine (Gilbert-Diamond et al. 2016). In one study, U-tAs was not associated with low birth weight but urinary monomethyl arsonic acid (MMA) was associated (22).

The effects of As exposure on birth outcomes may be mediated by the metabolic profile of the pregnant women which is observed in proportion of various As species (Milton et al. 2017). Nutritional factors, such as folate deficiency, can also directly affect As metabolism and potentially As-related effects (Laine et al. 2018), so factors as folates status and B12 vitamin should be considered for further studies. Other factor that could influence is the ingestion of arsenic through food, which is mainly present in rice-based foods which could lead to health risks in later life (Cubadda et al. 2017); nonetheless, in a cohort study of 1616 of Bangladeshi pregnant women, no association was found between rice consumption and toenail arsenic concentration (Lin et al. 2017), similarly in a New Hampshire population rice was not associated but other foods as dark meat fish and white wine were associated (Cottingham et al. 2013).

Tacna diet is based on carbohydrates, especially rice, but it is grown in the northern valleys of Peru, in Tumbes, being later distributed to the country. According to a recent study, it was found that 7 out of 29 Tumbes’ raw rice samples presented arsenic levels > 200 μg/L (Mondal et al. 2020). Tacna’s agricultural production is based mainly in olives (~ 50%) followed by pastures forage (~ 30%) (Dirección de Estadística Agraria 2020), but no arsenic content analysis in these crops has been performed.

One major limitation is concerning about dietary intake of seafood/fish since it was not evaluated in our study; intake of these foods may increase U-tAs due to non-toxic organic arsenical compounds as arsenobetaine (Jones et al. 2016), generating an overestimation bias. This is overcome in the present study since we have included data on urinary inorganic arsenic.

Arsenic may impact our population differently to other places given its ethnic characteristics. Tacna is a region with remarkably high birth weights, the highest in Peru (Fano et al. 2019a), which appears attributable to its large percentage of Aymara ethnicity (Fano et al. 2019a), a group that usually presents this trait (Rothhammer et al. 2015). Our sample exhibited a self-identification as Aymara in 56% of the cases (Fano et al. 2019b) but ethnicity was not evaluated as covariate since there could be an underestimation given the negative perception of ethnicity.

Additionally, beneficial genetic polymorphisms in the gene *as3mt* that are associated with more favorable As metabolism (evidenced by a higher concentration of the less toxic metabolite DMA) (de la Rosa et al. 2017; Engström et al. 2011) have been found in the Aymara population.

Other genes and its polymorphisms related to the one-carbon metabolism are found to affect the physiological response of the individual against arsenic exposure, such as the N-6 adenine-specific DNA methyltransferase 1 (*N6AMT1*) (Harari et al. 2013), and should be considered in further studies. Finally, we could have had errors in recall in data collection where information had to be obtained from the mother because of insufficient medical records. Despite our studies' limitations, our study does present important novel data on U-tAs and U-iAs exposure in the pregnancy Tacna population.

## Conclusions

In summary, we found no association between U-tAs and U-iAs with birth outcomes but a suggestive dose–response effect on SGA. However, this effect must be further evaluated with other maternal outcomes and covariates including pre-eclampsia, gestation progression, and metabolics and genetics of the mother as it relates to arsenic metabolism. Also, a larger sample size may be necessary to achieve a more robust analysis.

## References

[CR1] Almberg KS, Turyk ME, Jones RM, Rankin K, Freels S, Graber JM, Stayner LT (2017). Arsenic in drinking water and adverse birth outcomes in Ohio. Environ Res.

[CR2] Alves da Cunha AJL, Sobrino Toro M, Gutiérrez C, Alarcón-Villaverde J (2017). Prevalence and associated factors of macrosomia in Peru, 2013. Rev Peruana Med Exp Salud Publ.

[CR3] Bloom M, Neamtiu I, Surdu S, Pop C, Anastasiu D, Appleton A, Gurzau E (2016). Low level arsenic contaminated water consumption and birth outcomes in Romania-an exploratory study. Reprod Toxicol.

[CR4] Brammer H, Ravenscroft P (2009). Arsenic in groundwater: a threat to sustainable agriculture in South and South-east Asia. Environ Int.

[CR5] Castro De Esparza, ML (2006) *Presencia de arsénico en el agua de bebida en América Latina y su efecto en la salud pública*. Ciudad de México. Retrieved from https://www.bvsde.ops-oms.org/bvsacd/cd51/arsenico-agua.pdf. Accessed 3 March 2020

[CR6] CDC, Nceh, DLS, & Od (2019) *Fourth national report on human exposure to environmental chemicals, Updated Tables, January 2019*. Atlanta. Retrieved from https://www.cdc.gov/nchs/nhanes/.

[CR7] Concha G, Vogler G, Lezcano D, Nermell B, Vahter M (1998) Exposure to inorganic arsenic metabolites during early human development. Toxicol Sci (Vol. 44). Retrieved from https://academic.oup.com/toxsci/article-abstract/44/2/185/1611532 10.1006/toxs.1998.2486 9742656

[CR8] Cottingham KL, Karimi R, Gruber JF, Zens MS, Sayarath V, Folt CL, Karagas MR (2013). Diet and toenail arsenic concentrations in a New Hampshire population with arsenic-containing water. Nutr J.

[CR9] Cubadda F, Jackson BP, Cottingham KL, Van Horne YO, Kurzius-Spencer M (2017). Human exposure to dietary inorganic arsenic and other arsenic species: state of knowledge, gaps and uncertainties. Sci Total Environ.

[CR10] de la Rosa R, Steinmaus C, Akers NK, Conde L, Ferreccio C, Kalman D, Smith MT (2017). Associations between arsenic (+3 oxidation state) methyltransferase (AS3MT) and N-6 adenine-specific DNA methyltransferase 1 (N6AMT1) polymorphisms, arsenic metabolism, and cancer risk in a chilean population. Environ Mol Mutagen.

[CR11] De Loma J, Tirado N, Ascui F, Levi M, Vahter M, Broberg K, Gardon J (2019). Elevated arsenic exposure and efficient arsenic metabolism in indigenous women around Lake Poopó, Bolivia. Sci Total Environ.

[CR12] Dirección de Estadística Agraria. (2020). *Tacna Serie Histórica Producción Agrícola 2009–2018*. Tacna. Retrieved from https://www.agritacna.gob.pe/gestores/estadistica/of_ol_estadidet_e/archivos/6856489533_3085631670.pdf. Accessed 1 June 2020

[CR13] Engström K, Vahter M, Mlakar SJ, Concha G, Nermell B, Raqib R, Broberg K (2011). Polymorphisms in arsenic(+III Oxidation State) methyltransferase (AS3MT) predict gene expression of AS3MT as well as arsenic metabolism. Environ Health Perspect.

[CR14] Estrada-Restrepo A, Restrepo-Mesa SL, Feria NDCC, Santander FM (2016). Maternal factors associated with birth weight in term infants, Colombia, 2002–2011. Cadernos de Saude Publica.

[CR16] Fano D, Vásquez-Velásquez C, Ramirez-Atencio C, Yucra S, Gonzales GF (2019). Reproductive outcomes in pregnant women and its association with arsenic contamination in drinking water, in a region characterized by high birth weight rates in Peru. J Matern Fetal Neonatal Med.

[CR15] Fano D, Vásquez-Velásquez C, Aguilar J, Gribble MO, Wickliffe JK, Lichtveld MY, Gonzales GF (2019). Arsenic concentrations in household drinking water: a cross-sectional survey of pregnant women in Tacna, Peru, 2019. Exposure Health.

[CR17] George CM, Sima L, Arias MHJ, Mihalic J, Cabrera LZ, Danz D, Gilman RH (2014). Arsenic exposure in drinking water: an unrecognized health threat in Peru. Bull World Health Organ.

[CR18] Gilbert-Diamond D, Emond JA, Baker ER, Korrick SA, Karagas MR (2016). Relation between in Utero arsenic exposure and birth outcomes in a cohort of mothers and their newborns from New Hampshire. Environ Health Perspect.

[CR19] Hall M, Gamble M, Slavkovich V, Liu X, Levy D, Cheng Z, Graziano J (2007). Determinants of arsenic metabolism: blood arsenic metabolites, plasma folate, cobalamin, and homocysteine concentrations in maternal-newborn pairs. Environ Health Perspect.

[CR20] Harari F, Engström K, Concha G, Colque G, Vahter M, Broberg K (2013). N-6-adenine-specific DNA methyltransferase 1 (N6AMT1) polymorphisms and arsenic methylation in Andean women. Environ Health Perspect.

[CR21] Instituto Nacional de Estadística e Informática (2018) *Tacna Resultados Definitivos—Tomo IV*. Retrieved from https://www.inei.gob.pe/media/MenuRecursivo/publicaciones_digitales/Est/Lib1564/. Accessed 10 March 2020

[CR44] International Agency for Research on Cancer (2012) *Arsenic, metals, fibres and dusts, monograph volume 100C*. Lyon, France. Retrieved from https://monographs.iarc.fr. Accessed 25 February 2020

[CR22] Jones MR, Tellez-Plaza M, Vaidya D, Grau M, Francesconi KA, Geossler W, Navas-Acien A (2016). Estimation of inorganic arsenic exposure in populations with frequent seafood intake: evidence from MESA and NHANES. Am J Epidemiol.

[CR23] Kiserud T, Piaggio G, Carroli G, Widmer M, Carvalho J, Neerup Jensen L, Platt LD (2017). The World health organization fetal growth charts: a multinational longitudinal study of ultrasound biometric measurements and estimated fetal weight. PLOS Med.

[CR24] Kwok RK, Kaufmann RB, Jakariya M (2006). Arsenic in drinking-water and reproductive health outcomes: a study of participants in the Bangladesh integrated nutrition programme. J Health Popul Nutr.

[CR25] Laine JE, Ilievski V, Richardson DB, Herring AH, Stýblo M, Rubio-Andrade M, Fry RC (2018). Maternal one carbon metabolism and arsenic methylation in a pregnancy cohort in Mexico. J Eposure Sci Environ Epidemiol.

[CR26] Li DT, Liang Y, Gong YH, Chen MX, Feng P, Yang DG, Cheng G (2018). Relations between pregestational body mass index, gestational weight gain and birth weight of neonates among women in the Southwest areas of China: a prospective cohort study. Zhonghua Liu Xing Bing Xue Za Zhi Zhonghua Liuxingbingxue Zazhi.

[CR27] Liao KW, Chang CH, Tsai MS, Chien LC, Chung MY, Mao IF, Chen ML (2018). Associations between urinary total arsenic levels, fetal development, and neonatal birth outcomes: a cohort study in Taiwan. Sci Total Environ.

[CR28] Lin PID, Bromage S, Mostofa MG, Allen J, Oken E, Kile ML, Christiani DC (2017). Associations between diet and toenail arsenic concentration among pregnant women in bangladesh: a prospective study. Nutrients.

[CR29] Liu H, Lu S, Zhang B, Xia W, Liu W, Peng Y, Li Y (2018). Maternal arsenic exposure and birth outcomes: a birth cohort study in Wuhan, China. Environ Pollut.

[CR30] McClintock TR, Chen Y, Bundschuh J, Oliver JT, Navoni J, Olmos V, Parvez F (2012). Arsenic exposure in Latin America: biomarkers, risk assessments and related health effects. Sci Total Environ.

[CR31] Merklinger-Gruchala A, Jasienska G, Kapiszewska M (2015). Short interpregnancy interval and low birth weight: a role of parity. Am J Hum Biol.

[CR32] Milton AH, Hussain S, Akter S, Rahman M, Mouly TA, Mitchell K, Beale DJ (2017). A review of the effects of chronic arsenic exposure on adverse pregnancy outcomes. Environ Res Publ Health.

[CR33] Mondal D, Periche R, Tineo B, Bermejo LA, Rahman MM, Siddique AB, Cruz GJF (2020). Arsenic in peruvian rice cultivated in the major rice growing region of Tumbes river basin. Chemosphere.

[CR34] Mullin AM, Amarasiriwardena C, Cantoral-Preciado A, Henn BC, Leon Hsu H-H, Sanders AP, Burris HH (2019). Maternal blood arsenic levels and associations with birth weight-for-gestational age. Environ Res.

[CR35] Muñoz MP, Valdés M, Muñoz-Quezada MT, Lucero B, Rubilar P, Pino P, Iglesias V (2018). Urinary inorganic arsenic concentration and gestational diabetes mellitus in pregnant women from Arica, Chile. Int J Environ Res Public Health.

[CR36] Park SK, Peng Q, Bielak LF, Silver KD, Peyser PA, Mitchell BD (2016). Arsenic exposure is associated with diminished insulin sensitivity in non-diabetic Amish adults. Diab Metab Res Rev.

[CR37] Quansah R, Armah FA, Essumang DK, Luginaah I, Clarke E, Marfoh K, Dzodzomenyo M (2015). Association of arsenic with adverse pregnancy outcomes/infant mortality: a systematic review and meta-analysis. Environ Health Perspect.

[CR38] Rahman ML, Kile ML, Rodrigues EG, Valeri L, Raj A, Mazumdar M, Christiani DC (2018). Prenatal arsenic exposure, child marriage, and pregnancy weight gain: associations with preterm birth in Bangladesh. Environ Int.

[CR39] Ratnaike RN (2003). Acute and chronic arsenic toxicity. Postgrad Med J.

[CR40] Rothhammer F, Fuentes-Guajardo M, Chakraborty R, Lorenzo Bermejo J, Dittmar M (2015). Neonatal variables, altitude of residence and aymara ancestry in Northern Chile. PLoS ONE.

[CR41] Singh AP, Goel RK, Kaur T (2011). Mechanisms pertaining to arsenic toxicity. Toxicol Int.

[CR42] van de Bor, M (2019) Fetal toxicology. *Handbook of clinical neurology*, vol 162, Elsevier BV, Amsterdam, pp. 31–55 10.1016/B978-0-444-64029-1.00002-3 31324317

[CR43] World Health Organization (2011) *Arsenic in drinking-water background document for development of WHO guidelines for drinking-water quality*. Ginebra, Suiza. Retrieved from https://www.who.int/water_sanitation_health/dwq/chemicals/arsenic.pdf. Accessed 25 February 2020

[CR45] Xu L, Yokoyama K, Tian Y, Piao F-Y, Kitamura F, Kida H, Wang P (2011). Decrease in birth weight and gestational age by arsenic among the newborn in Shanghai China. Japan J Public Health.

